# High-intensity therapist-guided internet-based cognitive behavior therapy for alcohol use disorder: a pilot study

**DOI:** 10.1186/s12888-017-1355-6

**Published:** 2017-05-26

**Authors:** Christopher Sundström, Martin Kraepelien, Niels Eék, Claudia Fahlke, Viktor Kaldo, Anne H. Berman

**Affiliations:** 1 Department of Clinical Neuroscience, Karolinska Institutet & Stockholm Health Care Services, Stockholm County Council, Centre for Psychiatry Research, Norra Stationsgatan 69, -113 64 Stockholm, SE Sweden; 2 Stockholm Center for Dependency Disorders, 118 95 Stockholm, Sweden; 3 Department of Psychology, University of Gothenburg, Gothenborg, Gothenborg, Sweden

**Keywords:** Cognitive behavior therapy, Alcohol use disorders, Alcohol dependence, Internet-based psychotherapy

## Abstract

**Background:**

A large proportion of individuals with alcohol problems do not seek psychological treatment, but access to such treatment could potentially be increased by delivering it over the Internet. Cognitive behavior therapy (CBT) is widely recognized as one of the psychological treatments for alcohol problems for which evidence is most robust. This study evaluated a new, therapist-guided internet-based CBT program (entitled ePlus) for individuals with alcohol use disorders.

**Methods:**

Participants in the study (*n* = 13) were recruited through an alcohol self-help web site (www.alkoholhjalpen.se) and, after initial internet screening, were diagnostically assessed by telephone. Eligible participants were offered access to the therapist-guided 12-week program. The main outcomes were treatment usage data (module completion, treatment satisfaction) as well as glasses of alcohol consumed the preceding week, measured with the self-rated Timeline Followback (TLFB). Participant data were collected at screening (T0), immediately pre-treatment (T1), post-treatment (T2) and 3 months post-treatment (T3).

**Results:**

Most participants were active throughout the treatment and found it highly acceptable. Significant reductions in alcohol consumption with a large within-group effect size were found at the three-month follow-up. Secondary outcome measures of craving and self-efficacy, as well as depression and quality of life, also showed significant improvements with moderate to large within-group effect sizes.

**Conclusions:**

Therapist-guided internet-based CBT may be a feasible and effective alternative for people with alcohol use disorders. In view of the high acceptability and the large within-group effect sizes found in this small pilot, a randomized controlled trial investigating treatment efficacy is warranted.

**Trial registration:**

ClinicalTrials.gov (NCT02384278, February 26, 2015).

**Electronic supplementary material:**

The online version of this article (doi:10.1186/s12888-017-1355-6) contains supplementary material, which is available to authorized users.

## Background

It is well established that alcohol use has major detrimental effects on physical and mental health. Approximately 6% of all deaths in the world are ascribed to alcohol [1], currently the third leading risk factor for global disease burden [2]. While consumption levels are highest in Europe and North America, alcohol use is rapidly growing in developing countries such as China and India, with a resulting global overall increase in alcohol use during recent years [1]. Individuals with a diagnosed alcohol use disorder (AUD), i.e. those with impaired control over their alcohol consumption and who continue drinking despite serious adverse consequences [3], are estimated to account for about half of all global alcohol-related harm in developed countries [4]. In particular, the risk of dying from somatic diseases such as liver cirrhosis, cancer and cardiovascular disease is several times higher among people with an AUD [5]. The impact of AUD on morbidity and mortality is monumental, and increasing access to evidence-based treatment is imperative to health care.

Both pharmacological and psychological treatment alternatives for people with AUD can be considered evidence-based [3]. Among the better-known psychological evidence-based treatments are Motivational Interviewing [6] and cognitive behavior therapies such as Community Reinforcement Approach [7] and Relapse Prevention [8]. Although evidence-based treatments exist, few people with AUD seek help for their problems. For example, while recent epidemiological data indicated that 29% of adults in the USA had met the DSM-5 criteria for an AUD at some point in their lives, only 20% of these reported ever having sought help for their problems [9]. Similarly, a recent study conducted in a primary care setting in six different European countries found that only one in five people with current alcohol dependence had received professional help for their alcohol problems [10]. Research has identified several reasons why people with alcohol problems seek help to such a low extent. People may not believe that treatment will be effective, they may think that they should be able to deal with the problem on their own, and they may deny having a problem altogether; a further major barrier to seeking help is stigma [11]. In fact, fear of stigmatization has been shown to reduce the probability of seeking help for alcohol problems [12], a phenomenon that follows from the finding that AUD is the most severely stigmatized psychiatric condition of all. People with AUD provoke more social rejection and negative emotions than people with other psychiatric conditions, and are also held responsible for their condition to a greater extent than people suffering from depression and schizophrenia [13].

One way of increasing access to psychological treatment is to deliver it online. Advantages of doing this include cost-effectiveness and overcoming geographical barriers [14]. Delivering treatment online may also reduce the stigma associated with having to visit a clinic to receive treatment. There are two distinctly different ways of delivering psychological treatment online: programs available directly to the public, usually brief and without therapist contact but with some levels of automation, and more intensive programs guided by a therapist mirroring traditional manual-based face-to-face therapy [15] . Internet-based treatment delivered in this latter way is usually based on cognitive behavior therapy, and is then commonly referred to as ICBT [16]. This form of treatment has been developed and extensively studied with psychiatric disorders such as depression and anxiety [17], and there are examples of successful implementation of ICBT in regular care [18–20]. However, although potentially vast, the reach of ICBT remains quite limited and significantly increasing its accessibility is a challenge that has yet to be addressed [21].

Most of the online psychological treatments developed to target problematic alcohol use have been rather brief, low-intensity, automated open access programs without guidance from a therapist [22]. Although some of these programs have been based on cognitive behavior therapy [23, 24], most have focused on delivering ‘brief intervention’, a form of prophylactic single-session treatment typically aimed at helping hazardous drinkers moderate their drinking by providing screening and feedback on their alcohol consumption [25, 26]. Effects on alcohol consumption have generally been small [22], and a recent systematic review noted that the lack of internet interventions addressing AUDs specifically – not only hazardous drinking – hampers any clinical implications [27]. Internet-based treatment based on cognitive behavior therapy and guided by a therapist have, to our knowledge, been tested in three controlled studies [28–30]. Two of these studies evaluated the same treatment content [28, 29] which has been categorized as low-intensity [22]. The third study could be considered high-intensity as the therapist contact was quite extensive and participants were instructed to visit the program daily [30]. Moderate to large effect sizes on alcohol consumption were found in comparison to control groups in these studies (post-treatment 0.59 in Blankers et al. [28]; 0.75 in Sundström et al. [29]; 1.21 in Postel et al. [30]). Although these results are promising, none of the studies conducted standard interview-based diagnostic assessments with participants.

The aim of the present study was to evaluate the feasibility and preliminary effects of a high-intensity therapist-guided ICBT program for people with a diagnosed alcohol use disorder. We hypothesized that ICBT would be acceptable to the participants and that it would be associated with a reduction in alcohol consumption and in other alcohol-related measures.

## Methods

### Design

This was an uncontrolled pilot study intended to evaluate the feasibility and preliminary effects of a comprehensive, therapist-guided CBT program for alcohol use disorders. A within-group design with repeated measures was used.

### Procedure and participants

**Fig. 1:**
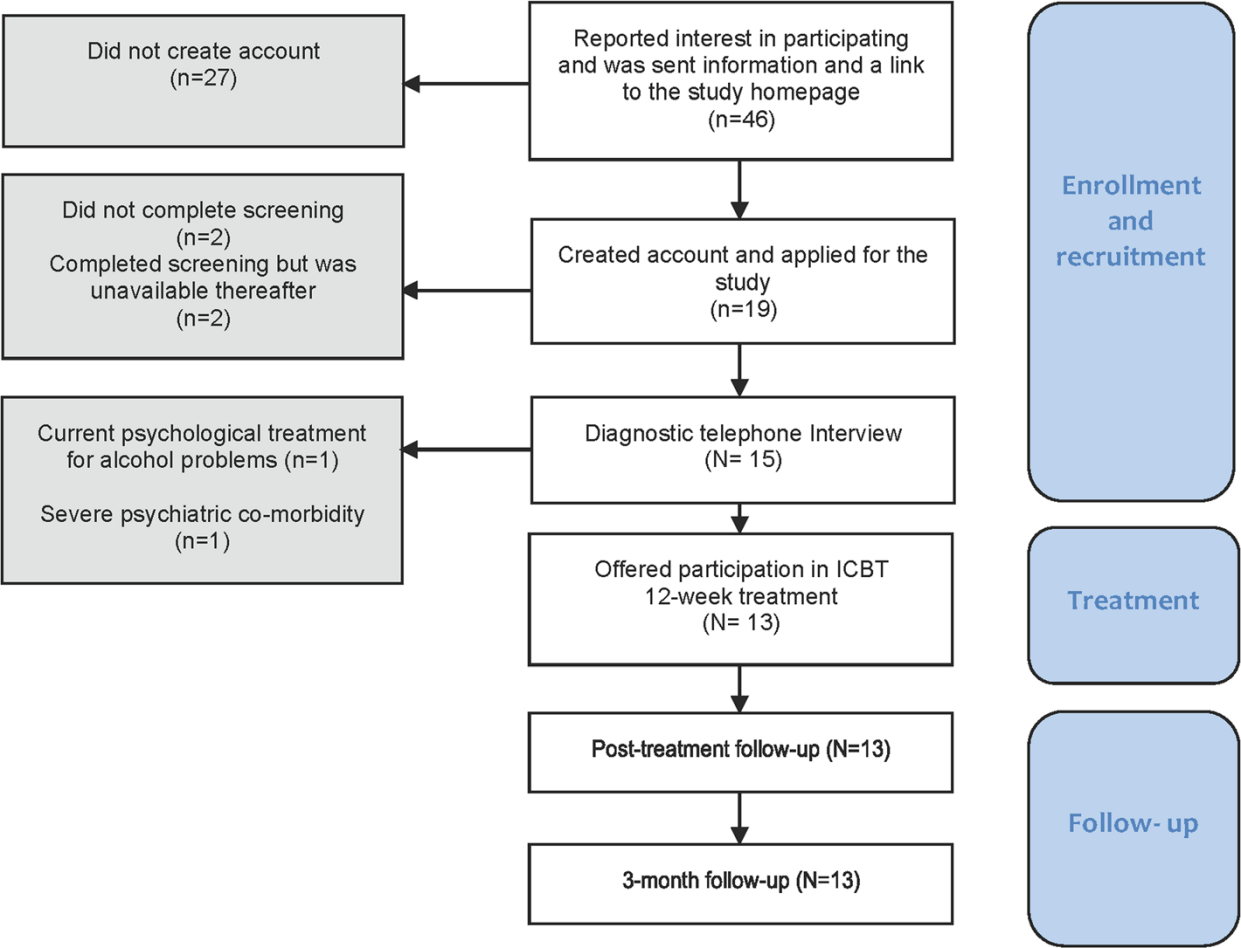
Participant flow chart

Between March 18 and April 13, 2015, an advertisement about the study was shown on the Swedish self-help site alkoholhjalpen.se. Individuals who registered their interest in participating were sent a brief presentation of the study along with a link to the program homepage where they could create an account in order to enroll in the study. The recruitment process was then conducted in two steps. The first step consisted of providing informed consent to participate in the study and completing online screening forms. The forms covered demographic characteristics (see Additional file 1) and eight questionnaires. The inclusion criterion in this first step was a score on the Alcohol Use Disorders Identification Test (AUDIT) [31] of 14 or more for women or 16 or more for men, indicating harmful use or dependence [32]. Exclusion criteria were a) severe depression as measured by the Montgomery Asberg Depression Self-Rating Scale (MADRS-S) [33] (a score of >30); and/or b) suicidal ideation as measured by 5 or 6 points on the MADRS-S question measuring suicidal ideation (item 9). In the second step of the recruitment process, participants eligible for inclusion after the first step were contacted and interviewed via telephone by a licensed clinical psychologist or a clinical psychology student at the Master of Science (MSc) level. The interview lasted about 30–45 min and consisted of three parts: 1) a brief anamnestic interview about the participant’s relation to alcohol and experiences of it (see Additional file 2); 2) a semi-structured interview for assessing AUD with the Structured Clinical Interview DSM IV (SCID-IV) [34], where criteria were adapted to the DSM-5 [35]; and 3) a structured interview to assess psychiatric comorbidity with the Mini International Neuropsychiatric Interview (MINI) [36]. The modules on AUD and Substance Use Disorders in MINI were not used, since the SCID-IV interview module on AUD provides more detailed information. Exclusion criteria in the second step of the recruitment process were: a) severe depression as measured by MINI or the anamnestic interview; b) severe psychiatric comorbidity as measured by MINI or the anamnestic interview; c) suicidal ideation according to MINI or the anamnestic interview; or d) ongoing psychological treatment for alcohol problems. Before receiving access to the program, participants once again completed two measures: the Time Line Follow Back (TLFB) and MADRS-S as pre-treatment measures. The treatment phase lasted 12 weeks and included contact via secure messaging with the therapist. Participants were given consecutive access to the modules in the program after completing homework assignments in the module worksheets. Three licensed clinical psychologists (CS, MK, NE) acted as therapists for the participants and provided feedback within 48 h during weekdays. Throughout the treatment, MADRS-S was administered weekly. If participants scored 4 or more at any time on item 9, which measures suicidal ideation, a therapist contacted them. At the beginning of the last treatment week, the participants again completed the questionnaires, except for the Readiness to Change Questionnaire (RCQ), which was only administered at screening. Participants also completed the Client Satisfaction Questionnaire-8 (CSQ-8) and other questions concerning evaluation of the treatment (see Additional file 3) and questions about whether other forms of support for alcohol problems had been received during the 12 weeks. Participants were also interviewed by telephone by the first author (CS) to assess adverse events (see Additional file 4) through the following question: “Have you at any point during treatment experienced one or more unwanted events that you feel was caused by the treatment, or experienced one or more unwanted effects due to the treatment?” Three months after treatment was completed, participants completed the post-treatment questionnaires again. Participants were not referred to any other treatments after the participating in the internet-based program offered in this study; no additional information about any other treatment was offered. For the participant flow throughout the study, see Fig. 1.

Participant demographics and characteristics of the 13 included participants are presented in Table 1. Three individuals screened positive for several psychiatric comorbidities in the diagnostic interview: one for social anxiety disorder, generalized anxiety disorder and antisocial personality disorder, one for depression and antisocial personality disorder, and one for social anxiety disorder and agoraphobia. One individual (age 26) had 3 points on the Drug Use Disorder Identification Test (DUDIT).

**Table 1:** Participants’ demographic characteristics at baseline

Variable	Category	*n* = 13
Age	Age in years, m (sd)	49.5 (15.0)
	Range in years	24–71
Gender	Female	9 (69%)
Marital status	Married/living with a partner/in a relationship	8 (62%)
	Divorced	4 (31%)
	Single	1 (8%)
Educational level	College/university	8 (62%)
	Secondary school (grades 10–12)	3 (23%)
	Compulsory school (grades 1–9)	2 (15%)
Current employment in percent (%)	Full time (100%)	8 (62%)
	Part-time	2 (16%)
	Not employed	3 (23%)
Self-perceived economic situation	Very good	4 (31%)
	Good	4 (31%)
	Neither good nor bad	4 (31%)
	Bad	1 (8%)
	Very bad	0 (0%)
Years with self-reported alcohol problems	1–2 years	3 (23%)
	3–5 years	3 (23%)
	6–10 years	1 (8%)
	More than 10 years	6 (46%)
Previously sought help for alcohol problems	Yes	6 (46%)
Prior support received when seeking help	Psychotherapy	2 (33%)
	Alcoholics Anonymous	2 (33%)
	Medication	4 (67%)
Alcohol Use Disorder (DSM-5)	Mild (2–3 criteria)	1 (8%)
	Moderate (4–5 criteria)	4 (31%)
	Severe (6 or more criteria)	8 (62%)
Psychiatric comorbidity	Yes	3 (23%)
Readiness to Change (RCQ)	Pre contemplation	0 (0%)
	Contemplation	1 (8%)
	Action	12 (93%)

## Measures

### Primary outcome

The Timeline Followback (TLFB) is a method of measuring alcohol consumption in terms of standard drinks over a specified length of time [37]. Participants were asked about the number of standard drinks consumed during the preceding seven days. TLFB delivered via computer has been found to yield data that correlate highly with paper and pencil [38]. Test-retest evaluation of a web-administered 7-day version of TLFB showed an intra-class correlation of 0.67, deemed fair [39]. In addition to TLFB, participants registered their preceding calendar week alcohol consumption once a week throughout the treatment by answering the following two questions: 1) How many standard drinks did you have the preceding calendar week? 2) Over how many days were these standard drinks distributed?

### Secondary outcomes

The Alcohol Use Disorders Identification Test (AUDIT) is a well-established and widely used 10-item instrument for measuring alcohol consumption and signs of harmful use and dependence related to alcohol consumption [31]. The instrument renders a score between 0 and 40. A score of between 6 and 13 for women and between 8 and 15 for men is considered to denote hazardous use; a score of between 14 and 19 for women, and between 16 and 19 for men is considered harmful use. A score of 20 or above for both men and women is considered probable alcohol dependence [40]. The web-administered Swedish version has shown internal consistency reliability in terms of Cronbach’s α values at 0.80–0.93 [41].

The Alcohol Abstinence Self-Efficacy Scale (AASE) is a scale measuring self-efficacy related to one’s ability to abstain from alcohol. Several versions of this instrument have been developed. The version used in this study consisted of 12 questions regarding the capacity to abstain from alcohol in various situations of temptation [42].

The Penn Alcohol Craving Scale (PACS) is a 5-item instrument that measures craving. PACS has been shown to have good convergent and discriminant validity as well as excellent internal consistency (Cronbach’s α of 0.92) [43]. The instrument was translated into Swedish by the first and last authors (CS, AHB).

The MADRS-S is an instrument measuring depression. It consists of nine items, each measuring a different symptom on a seven-point scale with a total scale ranging from 0 to 54 [44]. The instrument has been found to have excellent test-retest reliability and excellent internal consistency as well as to correlate with other similar instruments such as Beck Depression Inventory [33]. The online version has been found to have the same psychometric properties as the paper-and-pen version [45] with a Cronbach’s α of 0.73–0.90 [46].

The World Health Organization Quality of Life Scale-abbreviated version (WHOQOL-BREF) consists of 26 questions measuring quality of life on four domains: physical, psychological, social and environmental. The items are measured on a Likert scale of 1 to 5. Scores for each of the four domains are transformed into a score on a scale of 0–100. In a general population sample in Denmark, scores were found to be between 67 and 82 on each domain scale [47]. The instrument has yielded fair to good results for internal consistency, with Cronbach’s α of 0.68–0.82 in multiple language versions [48]. A recent study validated different online versions of the instrument, and found that these generated the same Cronbach’s alpha at levels similar to paper-and-pencil versions [49].

### Additional instruments

The Drug Use Disorders Identification Test (DUDIT) is an 11-item questionnaire designed to assess patterns of drug consumption and drug-related problems [50]. The instrument renders a score between 0 and 44, with a score between 8 and 24 considered harmful use [51], and a score of 25 or above considered probable drug dependence [50]. The DUDIT has shown good internal consistency reliability for a web-administered Swedish version at Cronbach’s α = 0.86 [52].

The Readiness to Change Questionnaire (RCQ) is an instrument for measuring the respondent’s motivation for changing their relation to alcohol [53]. The instrument contains 12 questions that, taken together, place the respondent in the pre-contemplation, contemplation or action phases of the Trans-Theoretical model of change. The Swedish version has yielded excellent internal consistency (Cronbach’s α = 0.88) [54]. To the authors’ knowledge, there is no validation of the RCQ for online administration.

The Client Satisfaction Scale-8 is an eight-item instrument that measures satisfaction with treatment [55]. Scores range from 0 to 32 with higher scores indicating greater satisfaction. The instrument has been found to have high internal consistency (Cronbach’s α of 0.92). To evaluate acceptability more thoroughly, CSQ-8 was complemented with questions about how the program and the therapist contact was perceived by participants, as well as questions about whether any other form of treatment had been received during the 12-week period. Acceptability was further assessed with a semi-structured interview conducted over telephone by the first author (CS) to assess adverse events [56].

### Treatment content

The treatment program consisted of 13 text-based modules, comprising about 80 pages in total, and an alcohol diary. Each module included educational texts, practical exercises, quizzes and worksheets. Table 2 presents the content of the treatment modules. The program was written by two of the authors (CS, AHB) with inspiration from cognitive behavior therapy and relapse prevention approaches [57] . The program was adapted to the more comprehensive and demanding ICBT interventions typically used at the Internet Psychiatry Clinic, Stockholm [58] with consultative input from three other authors (MK, NE and VK). The adaptation consisted mainly of adhering to the Internet Psychiatry clinic format, but also consisted of aligning some of the work sheet content with pedagogical components, making them a bit more explicit and concrete in reference to specific ‘behaviors.’

**Table 2:** Summary of the treatment manual: purpose and homework assignments

Module	Purpose of module	Homework assignment
Module 1Alcohol Education	T o learn about the effects of alcohol on body and mind and about tolerance and abstinence	Questions pertaining to the text
Module 2 Pros and cons of drinking	To help the participant reflect about pros and cons of drinking	Make a decisional balance
Module 3 Goals and values	To learn the difference between goals and values, and why these are important to establish at the beginning of treatment	Set an alcohol consumption goal during treatment (abstinence or moderate drinking) Explore and formulate core values in life
Module 4 Analyzing risk situations	To learn what risk situations are, and how to analyze them	Complete a behavioral analysis of one’s own risk situations
Module 5 Dealing with craving	To learn about craving and ways of dealing with it	Make notes on how to deal with craving: who can you call when you feel craving?, what can you do to distract yourself,? where can you surf on your craving?
Module 6 Dealing with thoughts about alcohol	To learn about what thoughts commonly occur among people who have just begun changing their alcohol habits	Make notes on which thoughts about alcohol occur most frequently Make a situational analysis and choose which specific coping strategies to use when the thoughts appear
Module 7 Dealing with social situations	T o learn about why it can be hard to say no to alcohol in social situations	Practice saying no with a friend or in front of a mirror Write down answers to specific situations presented in the text
Module 8 Finding other activities	To learn about the ‘’reward trap” (using alcohol as a reward), and the importance of finding other meaningful activities	List activities to engage in that do not include alcohol Draw up a time schedule for doing them
Module 9 Problem solving	To learn about stress, how it is sometimes associated with alcohol use, and about problem solving as a technique	Go through problem solving and apply it step by step in at least one situation
Module 10 Negative thoughts and interpretation traps	To learn about negative thoughts and about coping strategies to deal with them, cognitive restructuring	Complete a behavioral analysis of negative thoughts and challenge these thoughts
Module 11 Seemingly irrelevant decisions	To learn about the importance of identifying small, seemingly irrelevant decisions that could lead to drinking	Make notes on a situation where irrelevant decisions were involved in one’s drinking
Module 12 Relapse plan	To learn about the concept of relapse, and predict situations that could make it harder to resist drinking	Formulate a relapse plan
Module 13 Life without alcohol problems	To summarize the treatment and look towards the future	Review the initial alcohol consumption goal formulated in Module 1 Set goals for the future, after treatment

### Data analysis

SPSS version 22 (SPSS, Chicago, IL) was used for all statistical analyses. Descriptive statistics were calculated to summarize demographic characteristics at screening. To compare the preliminary efficacy of the treatment across time, generalized estimating equations (GEE) with an unstructured correlation matrix were run comparing primary and secondary outcome measures from screening to post-treatment, from screening to 3-month follow-up and from post-treatment to 3-month follow-up. On all outcome measures a normal model was used, apart from TLFB, where a negative binomial model was used due to the assumed non-normal distribution of alcohol consumption [59]. Data were analyzed according to the intention to treat (ITT) principle. Cohen’s d was used to calculate within-group effect sizes [Available, from:, http://www.cognitiveflexibility.org/effectsize/]. Since this was a pilot study intended to measure feasibility and preliminary effects, no power calculation was performed.

## Results

### Attrition

Two participants terminated treatment during the first week, one because of simultaneous enrollment in a group treatment for alcohol problems and one due to perceived lack of time. A third participant dropped out of the treatment about halfway through, due to self-reported stress and anxiety associated with partaking in the treatment. All three individuals were retained in the primary analysis according to the intention-to-treat (ITT) principle.

### Outcomes and effect sizes

Outcomes and effect sizes for alcohol consumption the preceding week and other outcome measures at screening, post-treatment and 3-month follow-up are presented in Table 3. The GEE analyses revealed significant main effects for time on all measures; pairwise comparisons are shown in Table 3.

**Table 3:** Observed mean (SE), change values (SE) and effect sizes

	Screening	Pre treatment	Post treatment	3 month follow up	Change screening to post-treatment	d	Change screening to 3-month follow up	d	Change posttreatment to 3- month follow- up	d
TLFB	23.4 (4.2)	19.5 (5.3)	10.3 (3.0)	5.1 (2.2)	13.1 (3.6)^c^	1.00	18.0 (4.4)^c^	1.20	4.9 (2.7)	0.54
AUDIT	23.7 (1.4)	-	14.4 (1.9)	10.9 (2.2)	9.3 (2.0)^c^	1.24	12.8 (2.0)^c^	1.82	3.5 (2.2)	0.42
AASE	2.19 (0.20)	-	3.39 (0.27)	3.65 (0.23)	1.20 (0.24)^c^	1.36	1.46 (0.24)^c^	1.59	0.26 (0.27)	0.25
PACS	13.3 (2.3)	-	9.5 (1.6)	5.3 (1.3)	3.9 (1.8)^a^	0.63	8.0 (1.8)^c^	1.32	4.2 (1.4)^b^	0.78
MADRS	14.9 (2.6)	12.9(1.8)	7.5 (1.8)	7.3 (1.9)	7.3 (2.7)^b^	0.75	7.5 (2.7)^b^	0.75	0.2 (1.2)	0.05
WHOQOL- Physical	63.2 (3.1)	-	72.0 (4.1)	74.5 (3.4)	8.8 (3.7)^a^	0.66	11.3 (3.6)^b^	0.83	2.5 (3.7)	0.18
WHOQOL- Psychological	53.8 (3.5)	-	66.7 (4.2)	67.6 (4.4)	12.8 (4.3)^b^	0.80	13.8 (4.9)^b^	0.76	1.0 (3.6)	0.07
WHOQOL- Social	54.5 (3.8)	-	64.1 (5.1)	67.3 (5.7)	9.6 (4.3)^a^	0.61	12.8 (5.4)^a^	0.66	3.2 (5.1)	0.17
WHOQOL-Environmental	72.8 (3.6)	-	77.2 (4.1)	79.1 (3.8)	4.3 (3.2)	0.37	6.3 (2.5)^a^	0.68	1.9 (2.1)	0.25

=^a^≤0.05, ^b^≤0.01, ^c^≤0.001

**Fig. 2:**
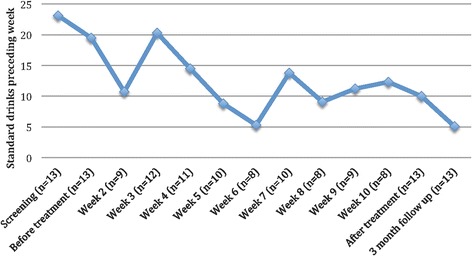
Mean number of standard drinks consumed preceding week during treatment

There was a mean time lag of 10.5 days between screening and start of treatment for the participants, ranging between 0 and 26 days. For this reason, and to control for some of the unspecific effects of participating in an assessment and taking the step of deciding to participate in treatment, effect sizes were also calculated for TLFB from Pre-treatment (rather than screening) to Post-treatment and the 3 month follow-up. The effect size for Pre-Post was 0.76 and for Pre-3-month follow-up was 0.79, which was lower than when using screening as baseline, but still significant (*p*-values = 0.02 and 0.007, respectively; not shown in Table 3). To illustrate the change process during treatment on the primary outcome, Fig. 2 shows the mean number of self-reported standard drinks over the preceding week during the treatment according to TLFB and weekly assessments. Concerning the DUDIT, at post-treatment three individuals (ages 26, 24 and 71) scored 3, 3 and 4 points respectively and at the 3-month follow-up two individuals scored 3 points each (not shown in Table 3).

### Treatment activity

During the 12-week treatment, the participants sent an average of 16.2 (sd = 7.2) messages to the therapist, ranging from 2 to 28 messages per participant. The therapists sent an average of 16.9 (sd = 10.4) messages to each participant, ranging from 1 to 35 messages per participant. Participants submitted an average of 59% of the 13 homework assignment report forms (m = 7.7, sd = 4.5) ranging from 0 to 13 homework report forms per participant.

### Treatment acceptability

Participant satisfaction ratings on the CSQ-8 indicated that satisfaction was excellent with a mean score on the CSQ-8 of 25.7 (range 18–32, SD = 3.98) [60]. Participants also answered evaluation questions specifically about their experiences of the treatment content, program usage, and the therapist contact. Concerning the treatment content, 91% stated that they found the text interesting and relevant, and all participants stated that they read 75% or more of everything there was to read. Concerning program usage, 73% stated that they worked quite actively with the homework assignments. Concerning therapist contact, 73% stated that they felt they had received lot of help from the therapist contact and 82% stated that they thought it was easy to understand what the therapist wrote.

### Other treatments and adverse events

One participant reported having initiated medication for alcohol use during the treatment (disulfiram), and four participants reported having received some other form of help for their alcohol use; two of these had visited a doctor, one had visited a self-help web site and one had attended 12-step meetings. No adverse events were reported in the post-treatment interviews. However, as mentioned previously, one participant did terminate treatment due to self-reported stress and anxiety, and although this participant did not define this as an adverse event in the interview, our clinical judgment is that it constituted an adverse event.

## Discussion

The aim of this pilot study was to investigate feasibility and preliminary effects of a comprehensive therapist-guided CBT-program for alcohol use disorders. Participants displayed high satisfaction with the treatment and most were active during the full treatment period; on average almost 60% of the modules were completed by the participants, fully comparable to what has been found in other studies on ICBT [61]. On average, a participant had slightly more than one contact with his or her therapist per week. Furthermore, a large within-group effect size was found on the primary outcome (standard drinks consumed preceding week) and moderate to large within-group effect sizes were found on the secondary outcomes of craving, self-efficacy, depression and quality of life. All improvements were maintained at the 3-month follow-up. In general, the effect sizes are in line with what has been found in other controlled trials investigating therapist-guided internet-based treatment for alcohol problems [28–30].

Internet-based interventions with therapist support have been found to be successful for several common psychiatric disorders and in some cases these interventions are already an integrated part of health care [62, 63]. Interventions for AUD, however, may entail specific challenges due to the potential long-term effects of alcohol on basic neuropsychological functions. Episodic memory deficits and executive dysfunctions have both repeatedly been associated with AUD early in abstinence [64, 65]. If internet interventions are to become an integral part of clinical care for people with AUD, a crucial aspect, after establishing effectiveness, would be to tailor interventions to patients with different neuropsychological profiles. Examples could be offering less text-heavy interventions to patients with episodic memory deficits or offering smart phone apps with automatic reminders to patients with executive dysfunctions [66]. Generally, clinical utility would be widened if neuropsychological aspects were taken into account when designing and developing the interventions. However, it is worth mentioning that about half of the participants in our study reported having had alcohol problems for more than 10 years, indicating that even people with a long history of severe problems can benefit from this form of intervention. More studies are needed to assess whether such tailoring is necessary and if so, under what diagnostic circumstances.

A significant strength of this study is the use of diagnostic assessment interviews conducted with participants to assess severity of alcohol problems. Few studies on internet-based interventions for alcohol problems have made any attempt at all to diagnose the participants, and, to our knowledge, those who did used self-administered online questionnaires, not interviews [30]. Diagnostic assessments facilitate generalizations to clinical populations, an important step if research results are to be translated into clinical practice. A further strength of this study is that we had follow-up data from all participants over all major time points. This is uncommon for studies on psychological interventions in general and for internet-based interventions in particular [67]. Compared to the previously mentioned therapist-guided internet treatment studies on alcohol, Postel et al. 2010 had an attrition rate of 54% [30], Blankers et al. 2011 had an attrition rate of 30% [28] and Sundström et al. 2016 had an attrition rate of 25% [29]. A probable explanation for the absence of attrition in this pilot study is that the diagnostic telephone interviews conducted before treatment made participants feel more obligated to participate in the follow-ups.

The purpose of this pilot study was not to evaluate causality, but to assess preliminary effects, acceptability and possible adverse effects. Use of a control group is always a prerequisite for establishing causality, and might be particularly important when attempting to establish efficacy of interventions for alcohol problems given the fact that so many people seem to be able to stop or reduce their drinking on their own without any or little help [68]. This important limitation should naturally be considered when interpreting the results. Furthermore, participants in our study were presumably highly motivated to change, since they were recruited through a self-help site. This high degree of motivation is reflected in the RCQ baseline scores, where almost all participants were in the ‘Action’ stage. Motivation is an important factor in all interventions for alcohol use disorders and the motivation among participants should be taken into consideration if the results are to be applied in clinical settings. Secondly, in our study, participants’ average baseline consumption of 23.1 standard drinks could be considered low. In other comparable studies, baseline consumption for preceding week has usually been somewhere between 30 and 45 standard drinks [23, 28, 30]. Alcohol consumption during the preceding week was not used as an inclusion criterion in our study, and three participants had an alcohol consumption during the preceding week below the recommended weekly limit of 9 standard drinks for women and 14 standard drinks for men at screening. The inclusion of these participants meant that there was little or no room for change in the primary outcome for these participants, thereby decreasing the effect size. It might also indicate that our study included participants with a lower severity of problems compared to other studies. However, several aspects indicate that our participants did have severe alcohol problems despite some of them having a low consumption the week before baseline measurement. The mean AUDIT score at baseline screening in our sample was well over 20, which is the cut-off for probable alcohol dependence, and our diagnostic interviews identified all of our recruited participants as having an AUD according to the diagnostic critieria (2 or more positive criteria) with eight of these (67%) having a severe AUD.

If the preliminary positive results in the current study could be confirmed in a randomized controlled trial, this would have important clinical implications. There are several potential advantages to offering comprehensive and effective treatment packages online for people with alcohol problems. As previously mentioned, it is well known that stigma and shame are common among people with alcohol use disorders, and may hinder seeking adequate help [69]. Online treatment could help reduce the treatment gap, by increasing access to treatment for people who find it difficult and anxiety-provoking to visit a treatment clinic and talk with a therapist about their alcohol problems. Other advantages with digital interventions include the logistical aspect. For example, countries such as Sweden and Australia are sparsely populated, and for people living in rural areas, visiting a clinic in person might mean having to undertake extended travels to receive help. After-care could also be handled with digital interventions, as has been shown in a recent publication where a mobile-phone app was successful in reducing number of risky drinking days [70]. Lastly, it is very common for alcohol treatment to only be offered in support groups. Although support groups are often appreciated, it is important that a variety of treatment options are available for this heterogeneous population.

## Conclusion

ICBT seems to be a feasible, safe and acceptable treatment for people with alcohol use disorders. The next step is to validate the effects in a randomized controlled trial.

## Additional files

Additional file 1: Screening questions. (DOCX 81 kb)

Additional file 2: Diagnostic interview. (DOCX 116 kb)

Additional file 3: Evaluation questions. (DOCX 90 kb)

Additional file 4: Evaluation and adverse effects interview. (DOCX 143 kb)
