# Supplementary material for: High-intensity therapist-guided internet-based cognitive behavior therapy for alcohol use disorder: a pilot study
Source: BMC Psychiatry. 2017 May 26;17:197. doi: 10.1186/s12888-017-1355-6 (PMC5446753; doi:10.1186/s12888-017-1355-6)
Supplement: Supplementary file 1 — Screening questions. (DOCX 81 kb) [file 12888_2017_1355_MOESM1_ESM.docx]

**Screening Questions**

1. **How did you find out about this study?**

- Ad on the internet
- Other, specify: _________________

1. **Gender**

- Male
- Female

1. **Age**

- ______ years

1. **What is your current relationship/marital status?**

- Married/Registered partner/Sambo/In a permanent relationship
- Divorced/Widow
- Single
- Other: ............................................. ....

1. **What kind of education do you have?**

- Elementary school
- High school
- University
- Other: _____________________________

1. **Current employment situation (fill in several options if needed)**

- Full time Employee/Entrepreneur /Student
- Sick-, sick-pensioner or the like
- Job-seeker
- Retired/early retirement
- Housewife/man
- Parental leave
- Other: ...........

1. **To what extent are you currently engaged in work, studies and or business?**

- 100%
- 75%
- 50%
- 25%
- 0%

1. **If you are engaged less than 100%, do you think that your alcohol problems are a cause of this?**

- No, my alcohol problems do not affect how much I'm engaged
- Yes, my alcohol problems affect to some extent how much I'm engaged
- Yes, my alcohol problems affect to a large extent how much I'm engaged
- Yes, my alcohol problem is absolutely crucial to how much I'm engaged

1. **What is your current financial situation like?**

- Very poor
- Poor
- Neither poor nor good
- Good
- Very good

1. **About how long do you think you have had alcohol problems?**

- Less than a year
- 1-2 years
- 3-5 years
- 6-10 years
- More than 10 years

1. **Have you previously sought help for your alcohol problems?**

- Yes
- No

1. **What kind of help did you receive? You can fill in several options.**

- Psychotherapy
- Alcoholics Anonymous / 12-step program
- Medication
- Have not received any help
- Other_____

1. **If the study seems to suit you, we will need to call you to make a telephone interview. Therefore, we ask you to enter a phone number we can reach you as well as times we can reach you.**

Telephone number_______

Times I can be reached______

Times I cannot be reached_____

1. **To participate in the study, you need time to engage with the treatment. Think about, for example, trips, demanding medical treatments, relocation to another place, major changes in your private or professional life or anything that requires time and power to prevent you from being treated.**

- Yes, I will be able to engage in alcohol treatment
- No, I will not have time
- I'm hesitant, but I would like to try and do my best

1. **In order for you to participate in the study you also need to master Swedish. The treatment is based on written material, and all questionnaires and forms are in Swedish. Telephone interviews are performed in Swedish only. Please mark with what is applicable to you.**

- Yes, I master Swedish
- No, I do not know Swedish
- I know Swedish, but not completely fluent
- My Swedish is not so good, but I can get help and support

1. **Finally, you need access to a computer with internet connection because the treatment itself and questionnaires are on the Internet. Please mark with one or more crossing what applies to you.**

- I have daily access to a computer with internet connection
- I have access to the Internet about 2-3 times a week
- I have Internet access once a week or less
- I do not have access to a computer with internet connection at all
- I have access to printer
