# Supplementary material for: High-intensity therapist-guided internet-based cognitive behavior therapy for alcohol use disorder: a pilot study
Source: BMC Psychiatry. 2017 May 26;17:197. doi: 10.1186/s12888-017-1355-6 (PMC5446753; doi:10.1186/s12888-017-1355-6)
Supplement: Supplementary file 2 — Diagnostic interview. (DOCX 116 kb) [file 12888_2017_1355_MOESM2_ESM.docx]

**Diagnostic interview**

Hi!

My name is ________________, I am a psychologist (psychology student) and am calling about the online study that you have signed up for. Thank you for showing an interest in participating! Before you join the study, we need to make an interview with you. This interview is about your relationship with alcohol and about your mood in general. It will take approximately 20-40 minutes. Do you have the opportunity to do this now? If not, when can I return? Do you have any questions before we get started? Ok! So, if we could start off by you telling me a bit about your relationship to alcohol.

(Make sure to get a brief description of the participant's story before you start going through the SCID criteria. The following questions are applicable: Has drinking led to any problems for you?; Was someone close to you complaining about your drinking?; How long has the have you had alcohol problems?; Have you been treated for alcohol problems earlier?; What kind of help did you receive?; For how long?)

**SCID-IV**

Use the SCID manual and mark each positive criterion with an X below

(Note! Skip E4, but instead ask about craving, see below):

- E2 Use leading to failure in important areas _____
- E3 Use when it is physically dangerous and risky______
- E5 Social/Interpersonal Problems_____
- E7 Used more/longer time than intended_____
- E8 Repeated attempts to stop/cut down ________
- E9 Much time spent on use__________
- E10 Giving up activities to use_____
- E11 Physical and mental problems_____
- E12 Tolerance _________
- E13 Abstinence _________

**Extra criteria for DSM5: Craving**

How often have you been thinking about drinking alcohol or how good you would feel if you drank alcohol? How strong has your craving for alcohol been when it has been the strongest? Have you taken a lot of time thinking about drinking alcohol or how good you would feel if you should drink alcohol?

- Craving ______

**Mini Neuropsychiatric Interview (MINI)**

Mark each positive diagnosis with an X below

- Depression ________
- Suicidality ________

(if suicidality is judged to be high, pay attention to this and check out 1177 to guide the participant to appropriate help)

- Manic/Hypomanic episode_____
- Panic Syndrome _______
- Agoraphobia________
- Social Phobia______
- Obsessive-compulsive disorder ____
- Posttraumatic stress syndrome____
- Psychotic Syndrome and Affective Syndrome with Psychotic Traits ___
- Anorexia Nervosa___
- Bulimia Nervosa____
- Generalized anxiety disorder ___
- Anti-social personality disorder ___

If the participant does not have high suicide risk, or PTSD, psychosis or bipolar disorder, the participant should be included in the study. Say: "Thank you! You are included in the study, and we will hear from you when the study starts via either SMS or phone calls." If the participant has any of the above diagnoses or if there are doubts about inclusion for other reasons, an assessment in the group needs to be done first. Say: "Thank you! I will return to you with information about whether you are included or not. "
