# Supplementary material for: High-intensity therapist-guided internet-based cognitive behavior therapy for alcohol use disorder: a pilot study
Source: BMC Psychiatry. 2017 May 26;17:197. doi: 10.1186/s12888-017-1355-6 (PMC5446753; doi:10.1186/s12888-017-1355-6)
Supplement: Supplementary file 3 — Evaluation questions. (DOCX 90 kb) [file 12888_2017_1355_MOESM3_ESM.docx]

**Evaluation questions**

**A. General questions about the treatment (questions 1-8 correspond to the Client Satisfaction Questionnaire mentioned under “Instruments”).**

1) How would you rate the quality of your treatment?

- Excellent
- Good
- OK
- Bad

Comment:______________________________________

________________________________________________

2) Did you get the kind of treatment you had wanted?

- No absolutely not
- No, not really
- Yes, basically
- Yes absolutely

Comment:______________________________________

________________________________________________

3) To what extent did treatment correspond to your needs?

- It corresponded to almost all my needs
- It corresponded to most of my needs
- It represented only a few of my needs
- It did not meet my needs at all

Comment:______________________________________

________________________________________________

4) If a good friend of yours would like similar help, would you recommend him / her the same kind of treatment you received?

- No absolutely not
- No I do not think so
- Yes, I think so
- Yes absolutely

Comment:_____________________________________

5) How satisfied are you with the amount of help you have received from us?

- Pretty unhappy
- Indifferent or slightly dissatisfied
- Mostly satisfied
- Very pleased

Comment:____________________________________

6) Has the treatment helped you to better address your problems?

- Yes, it has helped me a lot
- Yes, it has helped me something
- No, it did not really help me
- No, it seems to have worsened the situation

Comment:____________________________________

7) How satisfied are you IN TOTAL with your treatment?

- Very pleased
- Mainly satisfied
- Indifferent or slightly dissatisfied
- Really dissatisfied

Comment:_____________________________________

8) If you would like help in the future, would you come back to us?

- No absolutely not
- No I do not think so
- Yes I think so
- Yes absolutely

Comment:_____________________________________

**B. Questions about the content of the treatment**

How did the computer technology work?

- Very bad
- Badly
- Whether good or bad
- Good
- Very good

Was it too much or too little text?

- Far too little
- Too little
- Moderate
- Too much
- Too much

How much of all text did you read in total?

Nothing

- 25%
- 50%
- 75%
- 100%

How often did the text feel interesting and relevant to read?

- Never
- Rarely
- Sometimes
- Often
- Always

How easy or difficult was the text to understand?

- Very difficult
- Difficult
- Whether particularly easy or difficult
- Easy
- Very easy

How much of the text in each module did you read before you started working on your home assignments?

- Nothing or almost nothing
- Significantly less than half
- About half
- Significantly more than half
- Everything that was read

Was it easy or difficult to understand how you would work with your treatment and home assignments?

- Very hard
- Difficult
- Whether particularly easy or difficult
- Easy
- Very easy

Was it easy or difficult to understand how to fill in the worksheets?

- Very hard
- Difficult
- Whether particularly easy or difficult
- Easy
- Very easy

How much help did you think you received from your self-help (all but your personal contact with your therapist and the forum)?

- No help at all
- Not so much help
- Pretty much help
- A lot of help
- Very much help

How actively did you work with the different home assignments and tested what was addressed in the treatment? Do not count on how much you read about the treatment, but just how much you have done after reading.

- I did no homework at all
- I did not work so much at home with my homework
- I worked actively with my home assignments
- I worked actively with most home assignments
- I did all home assignments

What was your most beneficial treatment? Write what you had the most from the top.

I most benefited from: __________________________________________________

I had the second most benefit from: ______________________________________________

Then I most benefited from: ___________________________________________

**C. Contact with the therapist**

Did you manage the treatment as well without the therapist's support?

- I had managed treatment better without treatment support
- I had been treated equally well without treatment support
- I had managed treatment a little worse without treatment support
- I had managed treatment poorer without treatment support
- I had managed treatment much worse without treatment support

How much help, overall, did you think you got in touch with the therapist?

- No help at all
- Not so much help
- Pretty much help
- A lot of help
- Very much help

When you had problems or thought about something - how often did you ask the therapist about this?

- Never
- Just a few occasions
- About every other time
- Almost always
- Each time
- (Had no problems or concerns)

Are you satisfied with how quickly the therapist responded?

- Very displeased
- Dissatisfied
- Whether satisfied or dissatisfied
- Satisfied
- Very pleased

Was it easy or difficult to understand what the therapist wrote?

- Very hard
- Difficult
- Whether particularly easy or difficult
- Easy
- Very easy

Do you think it's important to have the same therapist throughout the treatment?

- Not important at all
- Not so important
- Important
- Very important
- Extremely important

What did you think was missing in contact with the therapist? ____________________________

Do you think the communication with the therapist has been superficial or personal?

- Very shallow
- Superficial
- Whether superficial or personal
- Personal
- Very personal

If you should go through the treatment again. How well do you think the following complement to self-help material would be?

- No help at all
- Not so much help
- Pretty much help
- A lot of help
- Very much help
- Continuous written communication over the Internet (as of now)
- 20 minute phone calls a week.
- 1.5 hour grouping a week with 6-8 patients and a therapist.
- 1.5 hours of grouping a week with 6-8 patients without the presence of a therapist.
- 45-minute individual call each week.
- Own proposal for drafting.

.........................................................................

**D. Summary questions**

31) How easy was access to and start treatment?

- Very hard
- Difficult
- Whether easy or difficult
- Easy
- Very easy

Have you been friendly and respectful?

- No absolutely not
- No
- Yes, but there were some flaws
- Yes
- Yes absolutely.

Was it too little or too much time to undergo treatment?

- Far too little
- Too little
- Moderate
- Too much
- Too much

How labor-intensive and comprehensive do you think the treatment has been?

- Very labor-intensive
- Laborious
- Right so laborious
- Not so laborious
- Not at all labor-intensive

Has the treatment been worth the effort it has made?

- No, definitely not
- No
- Yes, but pretty well
- Yes
- Yes absolutely.

Are you satisfied with how your treatment was terminated?

- Very dissatisfied
- Dissatisfied
- Whether satisfied or dissatisfied
- Satisfied
- Very pleased

(A) Did you complete the treatment without any major problems, or did you encounter obstacles or interrupted it prematurely?

- I completed the treatment and completed all modules without any significant problems
- I completed the treatment and completed all modules but encountered some problems
- I was left in treatment for the whole treatment period but he did not work with all modules because of different problems
- I canceled treatment on my own initiative
- The processor took the initiative to suspend treatment

(B) [SHOW FOR ALL ANSWERS WITHOUT FIRST I (a)].

If you interrupted and / or encountered problems, select one or more options that suits you and please write a comment under (c).

- I canceled prematurely because I was helped by the treatment and was pleased with the result
- I experienced that treatment did not help me
- I thought treatment was not about the issues I needed most help with
- I was too bad for time
- I did not feel like doing the treatment
- I was dissatisfied with the treatment
- I was dissatisfied with the contact with the therapist
- Other problems (described under c)

(C) Please write a comment even if you tick one of the predetermined options: ___________

What did you think about getting online?

- Very bad
- Badly
- Whether good or bad
- Good
- Very good

Internet treatment is based on cognitive behavioral therapy (CBT). If in the future you would need to seek help for the same problem you had now, would you prefer CBT (individually, in groups or as self-help) or any other type of treatment in the first place?

- I would rather want a different treatment than CBT
- I would probably do a different treatment than CBT
- It would not matter
- I would probably recommend CBT
- I'd rather have CBT

Improvement suggestions and other comments: ___________________________
