# Supplementary material for: High-intensity therapist-guided internet-based cognitive behavior therapy for alcohol use disorder: a pilot study
Source: BMC Psychiatry. 2017 May 26;17:197. doi: 10.1186/s12888-017-1355-6 (PMC5446753; doi:10.1186/s12888-017-1355-6)
Supplement: Supplementary file 4 — Evaluation and adverse effects interview. (DOCX 143 kb) [file 12888_2017_1355_MOESM4_ESM.docx]

**Evaluation interview and adverse effects**

You have now completed the internet treatment for alcohol problems. We in the research team wish to thank you very much for participating in the project and also for your contribution in evaluating the treatment. It is extremely important for us in the research team to know how the treatment has been experienced. That way, we can keep what's good and improve what's less good when we continue to do more studies in the future. Our goal is that to make this kind of treatment for alcohol problems part of regular healthcare, as is already the case with some other psychiatric problems.

I will now ask you some questions about how you experienced the treatment. Some of the questions may feel like repetitions of the evaluation questionnaire you already made on the internet, but some of the questions are new. The reason we also make a brief evaluation over the phone is that we want to be able to capture things that may be missed in the questionnaire you responded to and give participants an opportunity to express themselves more freely about the experience of the treatment.

1. **What did you think about getting psychological treatment via the internet? Was it what you expected when you signed up for treatment?**
2. **What part of the treatment felt most relevant to you?**
3. **What felt least relevant? Was there any part of the treatment that you experienced as unnecessary or irrelevant? For example, modules, home tasks, therapist support, etc. And what did you think about receiving text message reminders?**
4. **How was it to not be able to express yourself verbally to a therapist, but only in writing? What do you think would have been different if you would have seen a therapist physically? Would it have been better or worse?**
5. **An important part of the treatment was the so-called “ePlus model”, which describes the importance of reducing the consumption of alcohol both through managing life balance and by managing specific risk situations. Do you remember how the model looked? Was it relevant to your problem? There was also a video that described the model, what did you think about that? Do you think that more video clips in treatment would be a good idea?**
6. **If you experienced the treatment as positive, what would you need now when the treatment is over to keep the changes? For example, if someone were to check up on you in a month or three months to see how it goes? Would you like to have continued access to treatment without therapist support or with therapist support? What would you think of a mobile app that reminds you to log in to the treatment platform or that could provide you with summaries of what you have learned during the treatment?**
7. **During treatment, have you experienced one or more unwanted events that you consider to be due to treatment, or have you had one or more unwanted effects of the treatment? If yes, describe the unwanted event or unwanted effect, and indicate when these treatments / effects occurred, how often they occurred and how long each event / effect has lasted. How negative do you think this affected your well-being when they occurred? How negative do you think it affects your well-being today?**
8. **In summary, what was the best aspect of the treatment and what could be improved?**

Thanks! We will return to you with a follow-up measurement in about 3 months. You will get a text message when it's time, at the beginning of September.
